# The impact of religiousness on life satisfaction and anxiety level of the patients with depression disorders treated at the neuro-psychiatric center in riem, munich

**DOI:** 10.1192/j.eurpsy.2021.882

**Published:** 2021-08-13

**Authors:** E.D. Cindik Herbrüggen, B. Akdag

**Affiliations:** Psychosomatik/psychotherapie, Neuro-Psychiatric Center Riem (Neuro-Psychiatrisches Zentrum Riem), München, Germany

**Keywords:** Life satisfaction, Depression, Religiosity

## Abstract

**Introduction:**

Religious people suffer less from depression disorder than less or non-religious people. According to a longitudinal study investigating religiousness and negative life events, religious participants demonstrated fewer depressive symptoms than non-religious. Furthermore, depressed patients with higher religiosity scores show lower values of depression symptoms.

**Objectives:**

The purpose of the study was to investigate the relationship between religiosity and patients with depression symptoms in the Neuro-Psychiatric Center in Riem (NPZR). The correlation between religiousness and life satisfaction as well as anxiety level was analyzed. Additionally, possible gender differences are also assesed.

**Methods:**

The patients of the NPZR were selected as sample of the study (N =106, F=61, M=45). The participants were provided with three surveys including the life satisfaction questionnaire, state trait anxiety inventory and the Centrality Scale. A Pearson Correlation was conducted to investigate the association between life satisfaction, level of anxiety and religiousness. T-Test was carried out to find out the differences between female and male patients.

**Results:**

There was a positive relationship between religiousness and life satisfaction of the depressed patients (r = .608, p= .001). There was also a significant relationship between religiosity and anxiety level (r = - .548, p < .001). However there was no significant difference between male and female patients with regard to their religiousness (t= .149, p= .882).

**Conclusions:**

The findings indicate that while there is a significant relationship between life satisfaction, level of anxiety and religiousness of the patients, the gender of the patients has no impact on the religiosity of participants.

